# Influence of Dispersed Oil on the Remote Sensing Reflectance—Field Experiment in the Baltic Sea

**DOI:** 10.3390/s21175733

**Published:** 2021-08-25

**Authors:** Kamila Haule, Henryk Toczek, Karolina Borzycka, Mirosław Darecki

**Affiliations:** 1 Department of Physics, Gdynia Maritime University, ul. Morska 81-87, 81-125 Gdynia, Poland; h.toczek@wm.umg.edu.pl; 2 Department of Marine Physics, Institute of Oceanology of Polish Academy of Sciences, ul. Powstańców Warszawy 55, 81-712 Sopot, Poland; borzycka@iopan.pl (K.B.); darecki@iopan.pl (M.D.)

**Keywords:** dispersed oil, remote sensing reflectance, oil pollution, water quality, oil detection

## Abstract

Remote sensing techniques currently used to detect oil spills have not yet demonstrated their applicability to dispersed forms of oil. However, oil droplets dispersed in seawater are known to modify the local optical properties and, consequently, the upwelling light flux. Theoretically possible, passive remote detection of oil droplets was never tested in the offshore conditions. This study presents a field experiment which demonstrates the capability of commercially available sensors to detect significant changes in the remote sensing reflectance *R_rs_* of seawater polluted by six types of dispersed oils (two crude oils, cylinder lubricant, biodiesel, and two marine gear lubricants). The experiment was based on the comparison of the upwelling radiance *L_u_* measured in a transparent tank floating in full immersion in seawater in the Southern Baltic Sea. The tank was first filled with natural seawater and then polluted by dispersed oils in five consecutive concentrations of 1–15 ppm. After addition of dispersed oils, spectra of *R_rs_* noticeably increased and the maximal increase varied from 40% to over three-fold at the highest oil droplet concentration. Moreover, the most affected *R_rs_* band ratios and band differences were analyzed and are discussed in the context of future construction of algorithms for dispersed oil detection.

## 1. Introduction

The fates of oil spilled on the sea surface or leaking from underwater sources continue to be an ongoing topic of investigation and monitoring due to the extent environmental consequences of such events. Development of the models and methods for oil spill detection, as well as cleanup techniques, has led to a significant decrease of deliberate discharges. Over the past half-century, statistics for the frequency of medium and large spills (greater than 7 tons) from tankers have shown a downward trend. The yearly average recorded in the 2010s was 6.3 spills, which is less than a tenth of the average, 78.8, recorded in the 1970s [1]. In the extremely highly trafficked Baltic Sea, the total number of detected oil spills reduced from 763 in 1989 (upon the beginning of aerial surveillance of spills) through 472 in 2002, to 62 in 2018, pointing towards a steady decrease [2]. However, even statistically less numerous, and of smaller volume, oil spills continue to occur, and destructively affect the local and global environment, to name only a few: marine and near-shore fauna [3,4,5], marine flora [6], human health [7], and the seashore activities [8,9]. Oil spilled on the sea surface immediately starts passing along a series of chemical and physical processes known as “weathering”, with the degree and speed dependent on the type of oil and the environmental factors, including spreading, evaporation of volatile fractions, dissolution of low molecular weight PAHs (Policyclic Aromatic Hydrocarbons), emulsification, dispersion, biodegradation, etc., described in detail in [10,11,12]. While the majority of research focuses on the detection and monitoring of surface slick, much less attention is paid to the further stages of still ongoing weathering [13,14].

Currently, the oil spill response is based on the active and passive remote oil slick detection supported by various signal processing methods as well as oil spill modeling. Spaceborne detection techniques include extensively studied synthetic aperture radar (SAR) together with numerous classification algorithms [15,16,17,18] and radiometric (optical) imagery [19,20,21]. On the other hand, aerial surveillance for oil spill response is commonly equipped in side-looking airborne radars (SLAR) and laser fluorosensors, as well as the complementary thermal infrared sensors [2,22,23]. Both, the passive and active systems have their unique advantage and limitations; however, they never showed applicability to dispersed forms of oil.

Currently the fastest and most efficient methods combine the satellite SAR and airborne multispectral imagery to detect oil slicks and stable water-in-oil emulsions (containing 60–80% of water) [16,24]. However, monitoring of the fates of oil should not finish at the stage of water-in-oil emulsion. Remote detection limits, expressed in terms of the oil slick thickness, continue to shift as new methods are implemented [25]. Although satellite SAR cannot distinguish between thick slicks and a sheen, it can discriminate between thick stable emulsions and non-emulsified oil [26,27]. Nevertheless, sun glint-based spaceborne sensors have already demonstrated their capability to estimate oil slick thickness [28,29]. Still more precise are the airborne techniques which apply to the non-emulsified oil slicks covering the sea surface with a film of 0.05–3 mm thickness (e.g., passive microwave radar MWR-P, Optimare Systems, Bremerhaven, Germany) or even down to 0.05 µm for laser fluorosensors [30,31]. Laser-induced fluorescence was used to detect and classify the fresh and emulsified forms of hydrocarbons [32,33,34]. Fluorescence-based methods were also successful in underwater in situ detection of dissolved hydrocarbons [35,36].

Nevertheless, there are no commonly applied methods for the remote detection and monitoring of the remaining after-spill dispersed oil droplets. All techniques applied to oil slicks are based on the oil–water contrast in terms of the backscattered signal and its spatial or spectral distribution. Advanced oil spill models help predict the dynamics and evolution of an oil slick [14], assisting the scientists and the authorities in forecasting their trajectories in order to develop the best clean-up plans. Such models calculate the probable amount of oil in the water column at specified points of time [37]; however, the results highly depend on the model input data, the assumptions about the character of oil weathering processes, and a multitude of other factors [14]. The challenge of model execution in real time points toward the need for fast remote verification methods.

From the optical point of view, dispersed oil droplets modify the light propagation in seawater in the area of their occurrence, sometimes very significantly [38,39,40,41]; however, the character of such changes is completely unlike the oil slicks. Oil droplets do not cause sun glint, nor do they smooth the sea surface; also, their spreading properties and interfacial tension differ from the continuous oil film [42,43]. This is why their detection and monitoring need a different approach. New generation of sensors with constantly improving sensitivity combined with the appropriate experiment-based methods shall enable detection and monitoring of dispersed forms of oil in the near future [44].

This paper presents the results of a field experiment which demonstrates the change of intensity and spectral characteristics of the upwelling radiance *L_u_*(*λ*) in seawater polluted by various concentrations and different types of dispersed oil in comparison to the unpolluted one. We show that *L_u_*(*λ*) is sensitive to subtle changes caused by 1–15 parts per million (ppm) pollution of six types of oils: two crude oils, two marine gear lubricant oils, a cylinder oil, and a biodiesel. Afterwards, we discuss the possible consequences of the presence of dispersed oil for the retrieval of other seawater characteristics from the bio-optical models based on *R_rs_* band ratios and band differences. Furthermore, we point to the *R_rs_* ratios and differences most affected by dispersed oil, which can be useful in the outlook of dispersed oil detection.

## 2. Materials and Methods

This study was based on the measurements performed in the Southern Baltic Sea in April 2016 during a research ship cruise onboard the research vessel *Oceania* (Institute of Oceanology, Polish Academy of Sciences). The field experiment was designed in order to verify the possibility of the remote detection of dispersed oil. The remote sensing reflectance *R_rs_* of natural seawater was compared to the *R_rs_* of seawater polluted by dispersed oils in controlled conditions.

### 2.1. Description of the Tank

**Figure 1:**
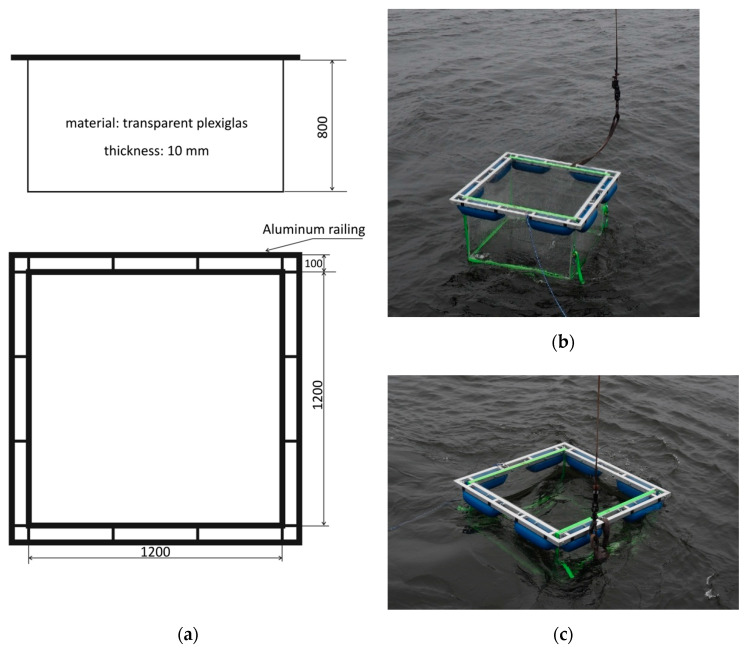
(**a**) Scheme of the floating tank for controlled assessment of the upwelling light in seawater polluted by dispersed oil droplets; (**b**) picture from field experiment illustrating placing the tank on the sea surface; (**c**) picture of the floating tank.

The oil-polluted area was limited to the dimensions of a transparent floating tank of 1.2 m × 1.2 m × 0.8 m, containing 1.152 m^3^ of water in the full immersion. Figure 1 shows the scheme of the tank and its implementation, first placed on the sea surface and then floating during the natural immersion.

The tank was made of transparent 2 cm thick blocks of plexiglass. Transparent walls ensured the maximum comparability of the experiment results with the natural light regime, modified only by the presence of dispersed oil. The construction of the tank allowed us to conduct *L_u_* measurements in almost natural conditions. The tank was strengthened on the top by a metal frame used to transport it from the ship deck onto seawater. The frame was also designed to hold the sensors inside the tank. Under the frame, eight cylindrical buoys were mounted to ensure the self-floating of the tank fully filled with seawater. The bottom of the tank had 12 round holes of 3 cm diameter each, designed for the fast self-filling of the tank after placing it on the sea surface. The leakage of dispersed oil through the holes during the measurements was negligible, considering the construction of the tank and its horizontal floating motion. Construction elements of the tank could potentially, but only to a minor extent, influence the measured upwelling radiance; however, their influence should be alike for unpolluted and polluted seawater. Such influence did not affect the result of this experiment and was considered as inessential, because the primary focus of this study was on the changes in the upwelling light caused by dispersed oil pollution.

**Figure 2:**
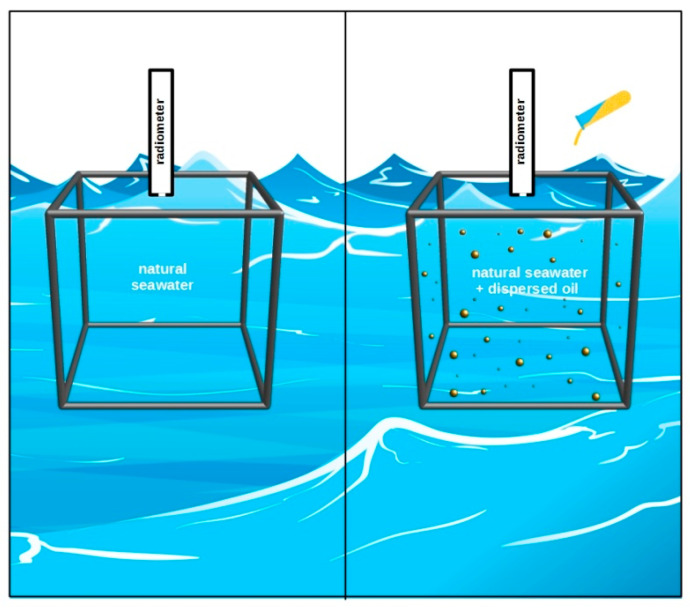
Pictorial drawing illustrating the concept of the field experiment. Hyperspectral remote sensing reflectance *R_rs_* measured in natural seawater was compared to the *R_rs_* in seawater polluted by dispersed oil droplets.

For the measurements, the tank was transported from the ship deck onto seawater. It was allowed to float awhile until it reached a position not affected by ship shadow. The scheme of the experiment concept is presented in Figure 2. Radiometric measurements were first carried out in the tank filled with natural seawater. Then, the tank was supplied with five consecutive portions of dispersed oil, and radiometric data were collected after each supplementation.

### 2.2. Optical Characteristics of the Background Natural Seawater

The Baltic Sea belongs to one of the basins most vulnerable to oil pollution. It is a very highly trafficked, semi-enclosed water basin with over 250 rivers, including nine large rivers flowing into it. The drainage area of the Baltic Sea is about four times larger than its surface area and is inhabited by around 85 million people [45]. Southern Baltic Sea coastal areas are rich in particulate organic matter from two major sources: autochthonous primary production and allochthonous riverine discharges [46]. Excess nutrient input is the main cause of eutrophication and, consequently, frequent algal blooms. This is why the inherent optical properties of the Baltic are measured on a regular basis, both in situ [47,48,49] and from space [50,51,52].

**Figure 3:**
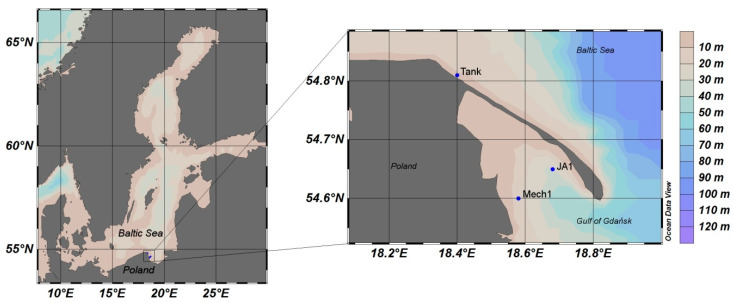
Field measurement locations in the Southern Baltic Sea.

**Figure 4:**
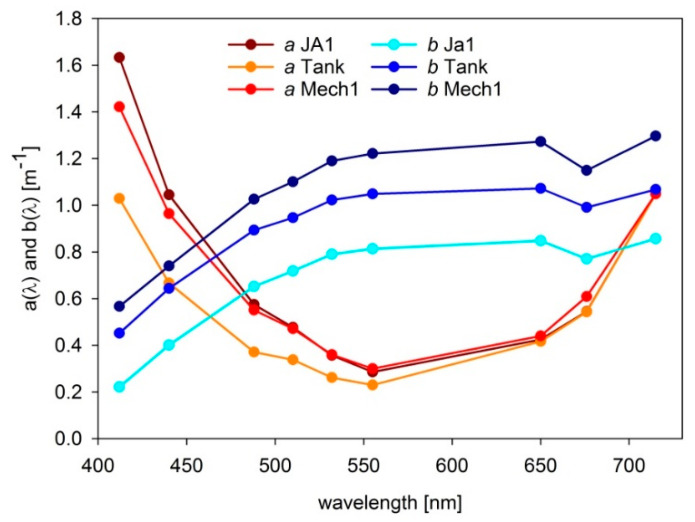
Spectra of absorption *a*(*λ*) and scattering *b*(*λ*) coefficients of the surface water layer of 0–1 m collected during the field campaign in April 2016 by AC-9 sensor at three stations: JA1 (13 April 2016), Tank (16 April 2016), and Mech1 (17 April 2016).

Our field experiment was conducted during a ship cruise of the *Oceania* *r*/*v* in the spring season on 13, 16, and 17 April 2016 at three stations: JA1 (N 54.65, E 18.68), Tank (N 54.81, E 18.40), and Mech1 (N 54.60, E 18.58). Locations of the stations are presented on the maps in Figure 3. Additionally, weather conditions and seawater characteristics from the stations are listed in Table 1. Figure 4 shows spectra of absorption *a*(*λ*) and scattering *b*(*λ*) coefficients collected in the surface layer (0–1 m) of natural seawater at the measurement stations using an absorption and attenuation meter AC-9 (WETLabs, Philomath, Oregon, USA). AC-9 data were registered with the frequency of six measurements per second, with the lowering speed 20–30 cm/s, which gives 20–30 measurements per each meter. Measurements were then interpolated to 1 m intervals from sea surface to sea bottom. Temperature and salinity corrections were applied to these measurements [53]. The signal was also corrected for scattering errors, which assumes zero absorption at 715 nm according to the recommended procedure [54].

Absorption coefficient at JA1 and Mech1, both placed in the Puck Bay, was comparable, as well as chlorophyll concentration level (see the last line in Table 1). The station Tank was placed in the open sea; therefore, the chlorophyll concentration and the absorption coefficient were accordingly lower. On the other hand, the highest scattering coefficient was at Mech1 and the lowest at JA1.

**Figure 5:**
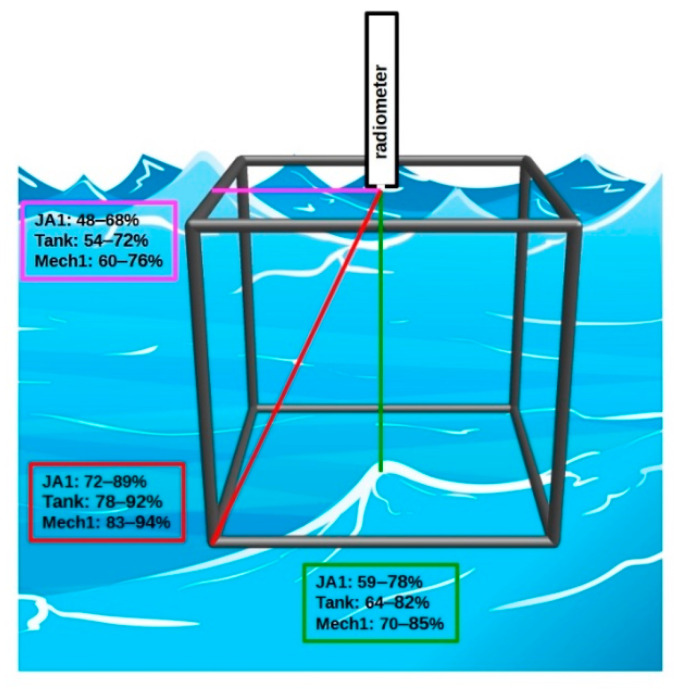
The fraction of the signal formed inside the tank at 555 nm (the lowest values) and 715 nm (the highest values) evaluated from the total attenuation coefficient *c*(*λ*) at three measurement stations: JA1, Tank, and Mech1 in three directions from the radiance sensor: horizontal of 0.6 m, vertical of 0.8 m, and the longest slant of 1.17 m.

Light attenuation coefficient *c*(*λ*) was used to estimate the fraction of measured light intensity coming from inside the tank, shown in Figure 5. It was estimated in three directions from the radiance sensor: horizontal of 0.6 m, vertical of 0.8 m and the longest slant of 1.17 m. Measured upwelling signal came from the tank interior in 48–76% from horizontal direction, in 59–85% from vertical direction, and in 72–94% from the longest slant. The lowest contribution was always related to 555 nm, which was the minimum of *c*(*λ*), and the highest to 715 and 412 nm—the maximum of *c*(*λ*). On average, about 60–90% of the measured upwelling signal originates inside the tank.

In the presence of additional absorbing and scattering components in the water, the measured signal is even more determined by the volume of water present in the tank, so from that point of view, we assumed that filling up the tank of the size described above with dispersed oil should sufficiently represent the upwelling radiance in the presence of dispersed oil in the natural conditions. The authors are aware that potential presence of dispersed oil outside the tank could make additional modifications of the light field within the tank, but these should be only minor and, if present, should only enhance the effect of dispersed oil on the *R_rs_*.

### 2.3. Preparation of Dispersed Oil Samples

Measurements were carried out for six types of oils. Their characteristics and their optical properties are summarized in Table 2. The oils included:

1. Crude oil *Petrobaltic* (PB), extracted offshore in the Southern Baltic in the Polish exclusive economic zone by LOTOS Petrobaltic S.A. (Gdańsk, Poland). PB is also known as *Rozewie* crude oil and it is extracted in majority from the B3 oil field located about 73 km north of Rozewie. It is characterized by about 73% of hydrocarbon content, an API gravity of 42–43, and a very low total sulfur content of 0.07–0.12 wt% [55]. PB belongs to light, very sweet crude oils.
2. Crude oil *Flotta Blend* (FL), extracted offshore in the North Sea in the British exclusive economic zone. It is a mixture of paraffin–naphthene-based hydrocarbons, characterized by an API gravity of 35–37, total sulfur content of 0.6–1.12 wt%, and total wax content of 6.75 wt% [55,56]. FL is a medium-heavy crude oil, significantly heavier than PB. Its sulfur content places it on the border between sweet and sour crudes, although it is more often referred to as a sweet or medium crude oil.
3. Cylinder lubricant oil *Cyliten N460* (CL), produced by LOTOS Oil S.A (Gdańsk, Poland). Its formula is based on >80% deeply refined, dewaxed, and hydrorefined mineral oils characterized by low susceptibility to coking, and greased with <20% vegetable oil for improving of the lubrication properties [57]. *Cyliten* is applied for lubrication of high-pressure compressors as well as low-speed gears, used, among others, in marine engine systems. It is distinguished by extremely high dynamic viscosity.
4. Biodiesel *BIO*-*100* (BD), purchased from PKN Orlen S.A. (Płock, Poland). It is a biofuel made of over 96% of fatty acid methyl esters. *BIO-100* is made from vegetable oils, usually rapeseed or sunflower oils. It is applicable for most diesel engines [58,59]. It is very bright to transparent by appearance and has extremely low viscosity.
5. Marine gear lubricant *Quicksilver Premium Gear Lube 80W-90* (QL), manufactured by Mercury Marine (Fond du Lac, WI, USA) for all kinds of outboards, recommended for use in marine gear cases with marine engines below 75 HP. It contains an emulsifier that improves protection of the gearbox against water ingress into the gear housing and additives improving the adhesion of the oil film.
6. Marine gear lubricant *Evinrude Johnson HPF*–*XR* (EJ) manufactured by BRP US Inc. (Sturtevant, WI, USA). It is the fill gearcase lubricant for two-stroke outboards. It is described as a high-viscosity blend of enhanced friction reducers, anti-foam agents, and synthetic extreme pressure additives.

**Figure 6:**
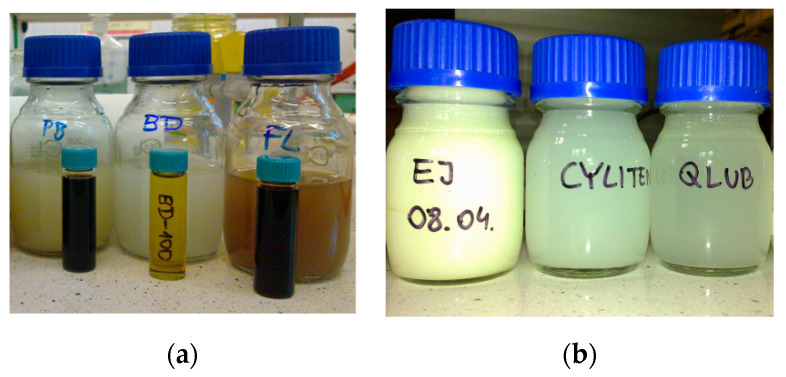
Samples of mechanically dispersed oils: (**a**) *Petrobaltic* PB, biodiesel BD, *Flotta* FL (small bottles contains pure oils, large bottles—concentrated oil dispersions); (**b**) *Evinrude Johnson* EJ, *Cyliten* CL, *Quicksilver Lube* QL.

Samples of mechanically dispersed oils were prepared onboard the ship 12–18 h prior to measurement, according to the procedure described in [44,60,61]. Obtained stable concentrated oil-in-water dispersions, pictured in Figure 6, were stored in glass bottles at room temperature.

Then, during field measurements, concentrated oil dispersions were poured into the floating tank filled with natural seawater in five consecutive portions in order to receive the intended final volume concentrations of 1 ppm, 3 ppm, 5 ppm, 10 ppm, and 15 ppm. Tank content was then mixed for 2 min by means of slow-speed mechanical stirring using a paddle. Maximal possible uniformity of oil distribution was ensured by thorough mixing, relatively low tank volume, and low oil concentrations.

**Figure 7:**
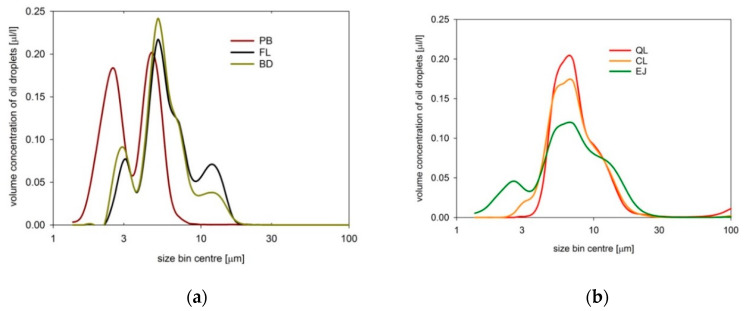
Size distributions measured by LISST-100X of oil-in-water dispersions normalized to 1 ppm for 6 types of oils: (**a**) *Petrobaltic* (PB), *Flotta* (FL), *BIO-100* (BD); (**b**) *Quicksilver Lube* (QL), *Cyliten N460* (CL), and *Evinrude Johnson Lubricant* (EJ).

Prior to the field experiment, we had measured droplet size distributions of oil samples prepared in the same way as during the field experiment, presented in Figure 7. Measurements were performed in April 2014 in the laboratory using a Laser In Situ Scattering and Transmissometer LISST-100X (type B, Sequoia Scientific, Inc., Bellevue, WA, USA) in a stationary mode. Droplet size distributions were registered in 3 s intervals in real-time operation mode. We collected a minimum of 100 scans for each measurement and averaged three repetitions for each sample of dispersed oil to minimize the heterogeneity uncertainties.

All oil dispersions consisted mostly of micrometer-sized droplets and reached main maxima between 5 and 7 µm. Dispersed BD, EJ, PB, and FL also contained smaller oil particles which contribute very significantly to the backscatter signal [62,63]. Dispersed EJ, QL, and CL (as well as FL and BD in a minor degree) also contained large oil droplets of tens of micrometers. Such droplets tend to absorb more light than small droplets.

### 2.4. In Situ Measurements of the Remote Sensing Reflectance R_rs_

The measurements carried out in the offshore conditions included the upwelling radiance *L_u_* measured just below sea surface and the downwelling irradiance *E_d_* measured by a reference sensor mounted on the ship deck. At every station, first *L_u_* was measured in the tank filled with natural seawater (see Figure 2). Next, the first portion of dispersed oil was poured into the floating tank and the content of the tank was stirred. The upwelling radiance was measured in the tank, and after each measurement, the optical windows of the sensor were cleaned. Then, the second portion of dispersed oil was poured into the floating tank, mixed, and *L_u_* data were collected again. This sequence was repeated for the third, fourth, and fifth portions of dispersed oil. At the end of each series of measurements, the tank was thoroughly washed and cleaned. Upwelling radiance measurements were averaged from a minimum of 430 measurements in order to minimize any heterogeneity and surface wave influence.

Upwelling radiance *L_u_*(0^−^,*λ*) (W m^−2^ nm^−1^ sr^−1^) just below the water surface was measured using the RAMSES−MRC (TriOS GmbH, Rastede, Germany) hyperspectral radiance sensor mounted on a special float, positioning the optical window approximately 2–5 cm below the sea surface. The head of the radiometer was custom designed with a narrow diameter in order to minimize the instrument self-shading effect. Simultaneously, downwelling irradiance *E_d_*(0^+^,*λ*) (W m^−2^ nm^−1^) above the water was measured using the RAMSES−ACC−VIS (TriOS GmbH, Rastede, Germany) hyperspectral irradiance sensor. To calculate the remote sensing reflectance, *L_u_*(0^−^,*λ*) was transferred into the air medium *L_u_*(0^+^,*λ*) by applying the immersion factor of Zibordi and Darecki [64]. Then, the remote sensing reflectance *R_rs_*(0^+^,*λ*) was calculated using the following equation:

(1) $$R_{rs}\left(0^{+},\lambda \right)=\frac{L_{u}\left(0^{+},\lambda \right)}{E_{d}\left(0^{+},\lambda \right)}$$

The variability of *L_w_* measurements was almost negligible because of the use of well-stabilized and calibrated radiometer and calm water conditions. Standard deviation did not exceed 2% in the analyzed spectral range.

## 3. Results and Discussion

### 3.1. Measurements of the Remote Sensing Reflectance

**Figure 8:**
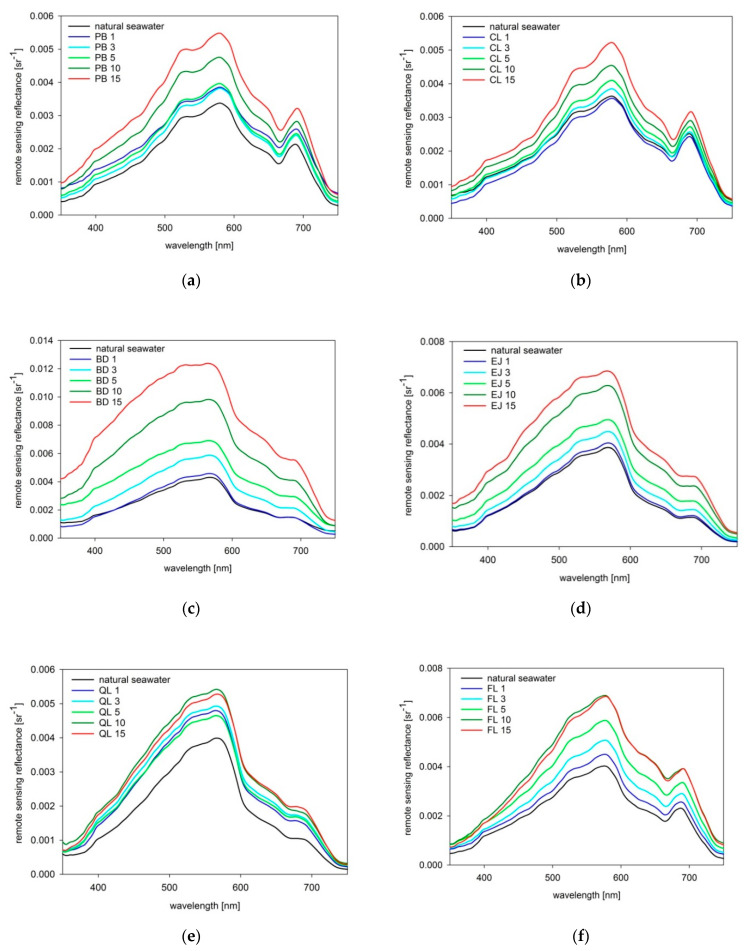
Remote sensing reflectance measured in natural seawater and seawater polluted by dispersed oil droplets in five consecutive concentrations for 6 types of oils: (**a**) *Petrobaltic* (PB); (**b**) *Cyliten N460* (CL); (**c**) biodiesel *BIO-100* (BD); (**d**) *Evinrude Johnson HPF-XR* (EJ); (**e**) *Quicksilver Lube* (QL); (**f**) *Flotta* (FL).

Results of the remote sensing reflectance *R_rs_* measured in the transparent tank filled with natural seawater and seawater polluted by dispersed oil droplets in five consecutive concentrations are illustrated in Figure 8. We observed a significant increase of the upwelling signal, especially clearly visible for high oil droplet concentrations of 10–15 ppm. Maximal *R_rs_* increase varied from about 40% for CL droplets to over three-fold for BD droplets.

Dispersed crude oil droplets have been already demonstrated as a substantial influence on optical characteristics of seawater in several radiative transfer modeling studies, e.g., [38,40,65]. Most of the past studies were limited to the analyses of one or two types of crude oils. The main conclusion of these studies was that at even as low a concentration as 1 ppm of oil, droplets can cause significant changes to the *R_rs_*. This effect was dependent on oil type [65], droplet size distribution [62], and seawater optical properties [61], as well as wind conditions and sensor geometry [66]. In this study, we took a step forward from modeling to in situ experiment in a unique attempt to measure the *R_rs_* of dispersed oil pollution in open sea conditions. We applied the idea of a comparative study between natural seawater and seawater polluted by oil droplets, as commonly practiced in ocean optics, e.g., on phytoplankton species [67] or other aquatic vegetation [68].

### 3.2. The Character of R_rs_ Changes Caused by Dispersed Oil Droplets

**Figure 9:**
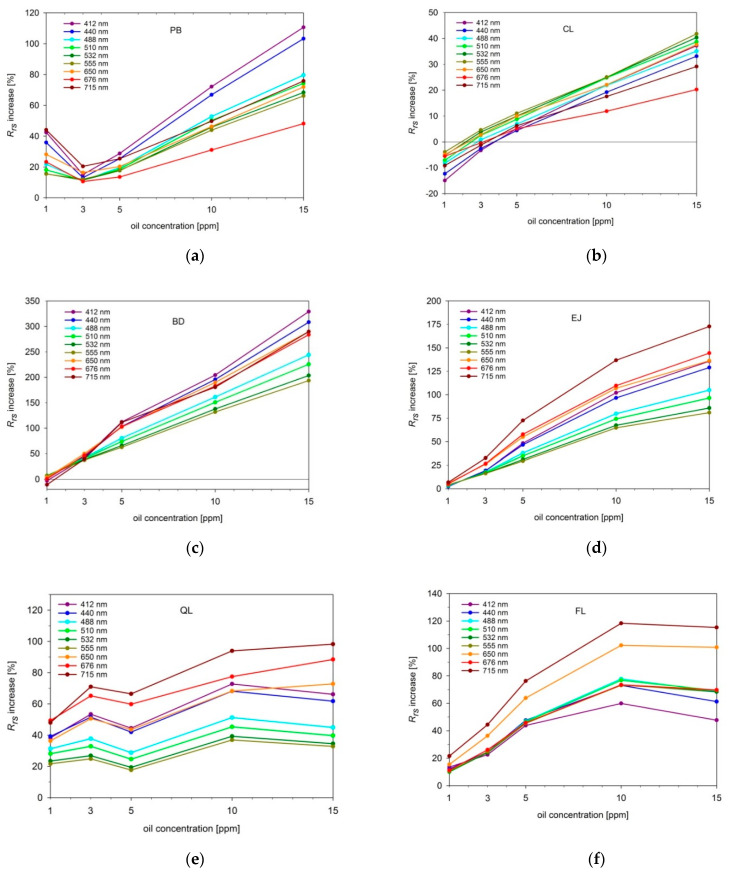
Percentage increase of the *R_rs_* plotted for 9 wavelengths (corresponding to AC-9 spectral bands) caused by dispersed oil droplets of (**a**) *Petrobaltic* (PB); (**b**) *Cyliten N460* (CL); (**c**) biodiesel *B-100* (BD); (**d**) *Evinrude Johnson HPF-XR* (EJ); (**e**) *Quicksilver Lube* (QL); (**f**) *Flotta* (FL).

Figure 9 displays spectral characteristics and the degree of changes of *R_rs_* caused by addition of five consecutive portions of dispersed oil droplets. PB and BD oil droplets (see Figure 9a,c) affected mostly the short (blue) visible waves of the upwelling light; CL droplets (see Figure 9b) most significantly increased the central (green) visible bands, while droplets of EJ, QL, and FL (see Figure 9d–f) resulted in the highest *R_rs_* increase at long (red) visible bands.

For some types of oils, *R_rs_* increases continuously proportional to the oil concentration (see Figure 9a–d). It should be noted that low oil concentrations of 1 and 3 ppm were characterized by higher relative uncertainties caused by limited oil dispersibility, deposition on walls, or heterogeneity of the mixed volume of water and oil. This is visible in Figure 8a where the *R_rs_* for 1, 3, and 5 ppm partially overlap, but when we compare the *R_rs_* for 5, 10, and 15 ppm we can see a steady increase, consistent with previous modeling results for similar droplet size distributions [38]. Another type of dispersed oil, CL (Figure 8b), decreased *R_rs_* at their lowest concentration, and then caused a steady increase at higher concentrations. A similar effect was observed in our previous modeling results for *Romashkino* crude oil, characterized by extremely high absorption coefficient which decreases the *R_rs_* [65]. Absorptive properties of CL are much lower than of *Romashkino*; however, they may be more significant at low droplet concentration than backscattering properties, which directly increases the *R_rs_*, and enhances with the growing number of tiny droplets. On the other hand, the gentle drop of *R_rs_* after adding the last oil portion, observed for QL and FL (Figure 8e,f), can be caused by spectral saturation. This might be connected to oil droplet coagulation, and thus explained by prevailing absorptive properties of larger droplets over backscattering properties [41,62].

### 3.3. Influence of Dispersed Oil on R_rs_ Band Ratios and Band Differences

**Figure 10:**
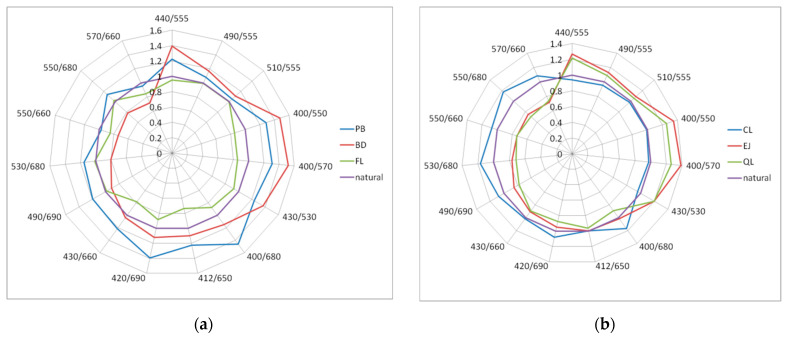
Polar plots of the impact of dispersed oils in the concentration of 15 ppm on selected *R_rs_* band ratios: (**a**) *Petrobaltic* (PB), biodiesel *B-100* (BD) and *Flotta* (FL); (**b**) *Cyliten N460* (CL), *Evinrude Johnson* (EJ) and *Quicksilver Lubricant* (QL). The scale shows values relative to the corresponding natural seawater band ratios.

The influence of dispersed oil on the selected *R_rs_* band ratios and *R_rs_* band differences is presented in Figure 10. Values of these factors, relative to natural seawater, are plotted for the maximal concentration of dispersed oil droplets of 15 ppm, and the following discussion refers to this concentration. Presented here is a brief analysis focusing on three types of *R_rs_* band ratios, described in the subsections below.

#### 3.3.1. “Blue-to-Green” *R_rs_* Ratios Typically Applied in Ocean Color Algorithms

Ocean color is the spectral variation of the water-leaving radiance that can be related to the concentrations of optically active constituents. Satellite detection of ocean color is commonly applied in global and local bio-optical algorithms in order to retrieve seawater characteristic properties, such as the concentration of chlorophyll-a or suspended particulate matter [69,70,71]. Most of the examined oils, except for CL and FL, increased the blue-to-green *R_rs_* ratios. Among NASA’s standard OCx algorithm band ratios [72], the highest increase was registered for the ratio of *R_rs_*(440)/*R_rs_*(555) (see the first three ratios on the polar plots in Figure 10). In particular, addition of dispersed PB, BD, EJ, and QL in the concentration of 10–15 ppm resulted in 20–40% increase of that ratio. On the other hand, CL oil droplets decreased that ratio by 6–9%, and FL oil droplets did not affect it in a significant way.

The next three ratios in Figure 10 illustrate other blue-to-green waveband combinations significantly affected by dispersed oils in our study. The ratio of *R_rs_*(400)/*R_rs_*(570) was the most sensitive to dispersed marine gear lubricant oils QL and EJ, as well as biodiesel BD and crude oil PB, and it increased by 26–52%. Dispersed biodiesel BD showed the greatest influence among all investigated oils on both *R_rs_* absolute values and band ratios. Results of measurements are in agreement with previously modeled data [38] for PB dispersions described by log-normal type of size distribution function, characterized by the peak diameter of 1 µm (when we compare absolute values of *R_rs_*) and characterized by the peak diameter of 0.5 µm (when we compare blue-to-green *R_rs_* band ratios).

#### 3.3.2. “Blue-to-Red” *R_rs_* Ratios

A further five ratios in Figure 10 represent blue-to-red *R_rs_* band ratios which do not have any standard global application in bio-optical algorithms in the ocean, however they are used locally in coastal waters, e.g., to derive surface concentrations of suspended particulate matter (SPM) and particulate organic carbon (POC) [71,73]. We found that there are *R_rs_* band ratios affected by all or most types of investigated oils. As an example, the ratio of *R_rs_*(490)/*R_rs_*(690) increased by 19.5% after addition of 15 ppm of PB, and decreased by 9–22% for BD, EJ, and QL. Similarly, the ratio of *R_rs_*(420)/*R_rs_*(690) was increased by 8–39% by some oils (CL, BD, PB) and decreased by others by 5–12% (EJ, FL, QL). Some particular *R_rs_* band ratios demonstrated the maximal influence for each type of oil droplets. Specifically, light crude PB oil droplets increased the ratio of *R_rs_*(400)/*R_rs_*(680) from 6% to almost 50% depending on droplet concentration. The maximal change caused by FL oil droplets was a 36% decrease observed for the ratio of *R_rs_*(412)/*R_rs_*(650).

Previous results of modeling carried out for dispersed *Petrobaltic* crude oil characterized by log-normal one-modal droplet size distribution [62] showed a reduction of blue-to-red *R_rs_* band ratios regardless of the size distribution peak diameter. In this experiment, the real field size structure of dispersed oils had a two-modal shape. On the other side, blue-to-red ratios decreased for FL, EJ, and QL, and their size distributions contain much more large (>10 µm) droplets. This is how we see that droplet size distribution plays a significant role in the remote detection of dispersed oils.

#### 3.3.3. “Green-to-Red” *R_rs_* Ratios

The last four *R_rs_* band ratios illustrated in Figure 10 represent green-to-red ratios, which are sometimes applied in bio-optical algorithms of complex waters, e.g., for estimation of chlorophyll-a concentration [70,71]. In this group of *R_rs_* ratios, we found some that may be potentially applicable for specific type of oil. Ratios *R_rs_*(550)/*R_rs_*(660) and *R_rs_*(570)/*R_rs_*(660) decreased by 36–40% for BD, EJ, and QL oil droplets and by 18% for FL, while the ratios *R_rs_*(550)/*R_rs_*(680) and *R_rs_*(530)/*R_rs_*(680) demonstrated 14–18% increase for PB and CL, and over 20% decrease for other oils. Marine lubricant oil droplets EJ and QL had the greatest effect on “green-to-red” *R_rs_* band ratios, reaching almost 50% for the ratio of *R_rs_*(532)/*R_rs_*(715). In addition, FL crude oil droplets noticeably increased that ratio by up to 30%. Oil influence on green-to-red ratios might be also dependent on natural seawater composition. Chlorophyll-a concentration was much lower at the Tank station, where the measurements were carried out for dispersed BD, EJ, and QL, and for these oils we noticed significant decrease of such ratios.

#### 3.3.4. *R_rs_* Band Differences

**Figure 11:**
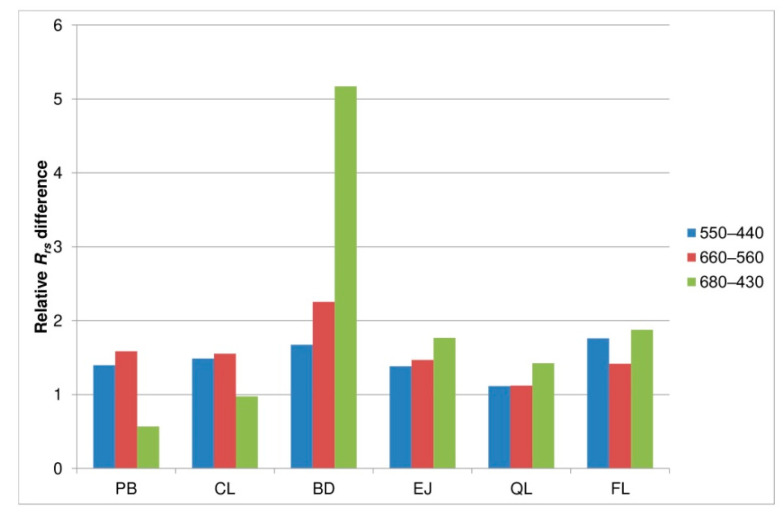
Relative *R_rs_* band differences of seawater polluted by dispersed oil droplets in the concentration of 15 ppm.

Furthermore, the analysis of three *R_rs_* differences is included and plotted in Figure 11. Considering high oil droplet concentrations (10–15 ppm), the “green–blue” difference of *R_rs_*(550)–*R_rs_*(440) increased from over 10% for CL oil droplets to over 70% for dispersed FL. The increase of the “red–green” difference of *R_rs_*(660)–*R_rs_*(560) varied from about 20% for QL oil droplets to 125% for BD oil droplets. The greatest effect was noticed for the “red-blue” difference of *R_rs_*(680)–*R_rs_*(430) reaching over a five-fold increase for dispersed BD, 88% for FL, and 77% for EJ. What is interesting is that PB oil droplets decreased that difference by 43% in comparison to natural seawater. Presented here, *R_rs_* band ratios and differences are good candidates for the structure of future algorithms for the remote detection of dispersed oil in seawater.

## 4. Conclusions

The first successful attempts of in situ measurements have been made in a self-designed floating tank. The promising results of this experiment provide an outlook for future research including continuation of in situ measurements and improving the model applicability on the basis of the field results. The field experiment was conducted during a cruise of the *Oceania r/v* in April 2016 in the Southern Baltic Sea. Remote sensing reflectance measured for natural seawater was compared to the one polluted by dispersed oils in controlled conditions. Data were collected for six types of oils. For most of the considered oils, there was a noticeable increase of the *R_rs_* values with oil droplet concentration. Maximal increase varied from about 40% for lubricant oil *Cyliten N460* (CL) droplets to over three-fold for biodiesel *BIO-100* (BD) droplets. The effect depended on oil type and on natural seawater composition. *Petrobaltic* (PB) and BD oil droplets mostly affected the short visible waves of the upwelling light, and CL droplets most significantly increased the central visible bands, while droplets of marine lubricants *Evinrude Johnson* (EJ) and *Quicksilver* (QL), as well as crude oil *Flotta* (FL), resulted in the highest *R_rs_* increase at long visible bands. Additionally, the impact of dispersed oils on *R_rs_* band ratios that are commonly used in ocean color and other bio-optical models was evaluated. Some “blue-to-green” ratios increased by up to 40%, while other ratios specific for each oil even indicated over a 50% increase. The field experiment provided a solid ground for future advancements and opens a new chapter for the remote sensing of dispersed oil.

## Figures and Tables

**Table 1:** Field experiment weather conditions and seawater characteristics.

Station	JA1	Tank	Mech1
Date	13 April 2016	16 April 2016	17 April 2016
Position	N 54.65, E 18.68	N 54.81, E 18.40	N 54.60, E 18.58
Measured oils	PB, CL	BD, EJ, QL	FL
Sky	Full overcast, diffusive conditions, drizzle	Clear sky (BD), single clouds (EJ), overcast (QL)	Full overcast, diffusive conditions, rain
Sea surface	Gentle to medium waves, thickly rough	Gentle waves, slightly rough	Gentle waves, slightly rough
Secchi depth	‒	5.5 m	4.5 m
Sea depth	78 m	11 m	12 m
Sea surface temperature	6.1 °C	5.7 °C	7.7 °C
Salinity	7.31–7.44 PSU	7.53–7.55 PSU	7.28 PSU
Chlorophyll concentration	8.91 mg/m^3^	2.36 mg/m^3^	11.49 mg/m^3^

**Table 2:** Characteristics and properties of oils selected for the marine experiment.

Mark	PB	FL	CL	BD	EJ	QL
Full Name	*Petrobaltic*	*Flotta*	*Cyliten 460N*	Biodiesel *BIO*-*100*	*Evinrude Johnson HPF*–*XR*	*Quicksilver Premium Gear Lube*
Type of oil	Light, very sweet crude oil	light, Sweet-sour crude oil	Mineral oil, cylinder lubricant	Biofuel	Mineral oil, marine gear lubricant	Mineral oil, marine gear lubricant
Main application	Energy industry	Energy industry	High-pressure compressors, low speed gears	Diesel engines	Motorboats, two-stroke outboards	Motorboats, all outboards
Dynamic viscosity in 20 °C, mPa·s	19.91	22.77	2140	4.86	183.5	164.2
Refractive index at 20 °C (400–700 nm)	1.4878–1.4649	1.5233–1.4909	1.5148–1.4918	1.4721–1.4523	1.4998–1.4797	1.5011–1.4805
Dispersion effectiveness *	30%	80%	86%	~100%	~100%	91%
Color	Dark brown with golden shade	Deep dark brown	Golden yellow	Yellow–green	Dark green	Dark red

* Estimated on the basis of the total volume concentration measured by the LISST-100X.

## Data Availability

The data presented in this study are available on request from the corresponding author.
